# Landscape of RNA-binding proteins in diagnostic utility, immune cell infiltration and PANoptosis features of heart failure

**DOI:** 10.3389/fgene.2022.1004163

**Published:** 2022-10-14

**Authors:** Jie Li, Xueqin Zhang, Peng Ren, Yu Wu, Yaoguo Wang, Wenzheng Zhou, Zhao Wang, Peng Chao

**Affiliations:** ^1^ Department of Cardiology, People’s Hospital of Xinjiang Uygur Autonomous Region, Urumqi, China; ^2^ Department of Nephrology, People’s Hospital of Xinjiang Uygur Autonomous Region, Urumqi, China; ^3^ Department of Medical Administration, People’s Hospital of Xinjiang Uygur Autonomous Region, Urumqi, China; ^4^ Department of Information Center, People’s Hospital of Xinjiang Uygur Autonomous Region, Urumqi, China; ^5^ Department of Orthopaedics, People’s Hospital of Xinjiang Uygur Autonomous Region, Urumqi, China

**Keywords:** heart failure, RNA binding protein, subtype, characteristic gene, nomogram, immune cells, PANoptosis

## Abstract

**Objective:** Heart failure remains a global public health problem linked to rising morbidity and mortality. RNA-binding proteins (RBPs) are crucial regulators in post-transcriptionally determining gene expression. Our study aimed to comprehensively elucidate the diagnostic utility and biological roles of RBPs in heart failure.

**Methods:** Genomic data of human failing and nonfailing left ventricular myocardium specimens were retrieved from the GEO datasets. Heart failure-specific RBPs were screened with differential expression analyses, and RBP-based subtypes were clustered with consensus clustering approach. GSEA was implemented for comparing KEGG pathways across subtypes. RBP-based subtype-related genes were screened with WGCNA. Afterwards, characteristic genes were selected through integrating LASSO and SVM-RFE approaches. A nomogram based on characteristic genes was established and verified through calibration curve, decision curve and clinical impact curve analyses. The abundance of immune cell types was estimated with CIBERSORT approach.

**Results:** Heart failure-specific RBPs were determined, which were remarkably linked to RNA metabolism process. Three RBP-based subtypes (namely C1, C2, C3) were established, characterized by distinct pathway activities and PANoptosis gene levels. C2 subtype presented the highest abundance of immune cells, followed by C1 and C3. Afterwards, ten characteristic genes were selected, which enabled to reliably diagnose heart failure risk. The characteristic gene-based nomogram enabled to accurately predict risk of heart failure, with the excellent clinical utility. Additionally, characteristic genes correlated to immune cell infiltration and PANoptosis genes.

**Conclusion:** Our findings comprehensively described the roles of RBPs in heart failure. Further research is required for verifying the effectiveness of RBP-based subtypes and characteristic genes in heart failure.

## Introduction

Heart failure is a frequent complex clinical syndrome of symptoms and signs triggered by structural or functional abnormality that leads to impaired cardiac output (Packer et al., 2021b), which remains a growing public health issue affecting about 26 million individuals globally (Heidenreich et al., 2022a). Typically, in accordance with left ventricular ejection fraction (LVEF), heart failure is categorized as preserved (HFpEF) and reduced ejection fraction (HFrEF) (Heidenreich et al., 2022b). HFpEF is a filling issue because of muscle stiffness reducing left ventricular chamber size or left atrial dilation, while HFrEF is usually described as a mechanical left ventricular pump issue (Mascolo et al., 2022). Therapeutic strategies of above two types differ. Substantial evidence suggests that sequential drug treatment improves clinical outcomes in patients with HFrEF (Berg et al., 2021). Differently, no treatment options show prognostic benefits and symptom controlling as the sole management regimen of HFpEF patients (Packer et al., 2021a). Reduction of associated hospital readmission rate and clinical and economic burden remains a pivotal issue in modern cardiovascular medicine (Piepoli et al., 2022). Biomarkers that reflect the pathophysiological processes of heart failure progression can aid clinicians in the early diagnosis and management of heart failure patients.

RNA binding proteins (RBPs) control RNA fate from synthesis to decay, the expression and roles of which are highly determined by detailed networks of transcriptional, post-transcriptional as well as post-translational machinery (Liu et al., 2022). They are implicated in pathological manifestations of heart failure. For instance, RBPs have widespread translational control of human cardiac fibroblast activation (Chothani et al., 2019). RNA binding protein 24 loss enables to disrupt global alternative splicing as well as results in heart failure (Liu et al., 2019). RNA-binding protein RBM20 weakens splicing to orchestrate cardiac pre-mRNA processing, and contributes to the pathogenesis of heart failure (Maatz et al., 2014). RBP HuR-mediated SCN5A mRNA stability represses arrhythmic risk in heart failure (Zhou et al., 2018). Knockdown of RNA binding motif-20-based titin splicing system can upregulate compliant titins, and thus ameliorates diastolic function and exercise tolerance in heart failure (Methawasin et al., 2016). Despite this, comprehensive analyses of RBPs in heart failure are lacking. Accumulated evidence demonstrates that PANoptosis (apoptosis, necroptosis, together with pyroptosis) mediates heart failure progression and possesses promising therapeutic implications (Zhang et al., 2016; Zeng et al., 2019; Gao et al., 2020). Nonetheless, the mechanisms of RBPs underlying PANoptosis remain indistinct in hear failure. Our study comprehensively evaluated RBP-based molecular subtypes and relevant characteristic genes for heart failure, unveiling the crucial roles of RBPs in pathophysiological process of heart failure as well as providing reliable targets for diagnosing heart failure risk.

## Materials and methods

### Heart failure expression profiling

This study downloaded the expression profiling of human heart failure from the Gene Expression Omnibus (GEO) repository. The GSE5406 dataset comprised microarray expression profiles of 194 human failing left ventricular (LV) myocardium specimens and 16 human nonfailing control LV myocardium specimens on the Affymetrix platform (Hannenhalli et al., 2006). Additionally, we acquired microarray expression profiles of 177 human failing LV myocardium specimens and 136 human control specimens from the GSE57338 dataset on the basis of the Affymetrix platform (Liu et al., 2015). Above expression profiles were merged, and removal of batch effects was implemented utilizing sva package (Leek et al., 2012). Four independent datasets were utilized as external verification sets as follows: the GSE76701 dataset comprising expression profiles of 4 non-failing and 4 failing LV hearts (Kim et al., 2016); the GSE55296 dataset containing RNA-seq data of human hearts from 26 heart failure patients and 10 healthy controls (Tarazón et al., 2014); the GSE86569 dataset with expression profiling of LV hearts from 12 HFrEF and 20 HFpEF patients; the GSE3585 dataset including expression profiles of 5 non-failing and 7 failing LV hearts.

### Screening heart failure-specific RBPs

Totally, 1,542 RBPs were collected from previously published literature (Supplementary Table S1) (Gerstberger et al., 2014). Expression values of RBPs were compared between human failing and nonfailing LV myocardium specimens through limma package (Ritchie et al., 2015). RBPs with adjusted *p* < 0.05 and |fold-change|>1.5 were regarded as heart failure-specific RBPs. Targets of RBPs were predicted through starBase database.

### Functional enrichment analyses

Gene Ontology (GO) and Kyoto Encyclopedia of Genes and Genomes (KEGG) enrichment analyses of RBPs with adjusted *p* < 0.05 were implemented utilizing clusterprofiler package (Yu et al., 2012). Terms with adjusted *p* < 0.05 were regarded as significant enrichment.

### Consensus clustering analyses

Through ConsensusClusterPlus package (Wilkerson and Hayes, 2010), on the basis of RBPs with adjusted *p* < 0.05, consensus clustering analyses of heart failure specimens were run in accordance with the following parameters: item resampling, proportion of items to sample: 80%; gene resampling, proportion of features to sample: 80%; a maximum evaluated k, maximum clustering number to evaluate: 9; resampling, number of subsamples: 1,000; agglomerative hierarchical clustering algorithm): ‘hc’ (hclust); and distance: ‘pearson’ (1 - Pearson correlation). The RBP-based subtype classification was verified through principal component analyses (PCA).

### Gene set enrichment analyses

GSEA methodology (Subramanian et al., 2005) was utilized for the comparisons of KEGG pathways across RBP-based subtypes. The “c2.cp.kegg.v7.4.symbols” gene set from the Molecular Signatures Database (Liberzon et al., 2015) was employed as a reference set, with the criteria of *p*-value<0.05.

### Weighted gene co-expression network analyses

Expression profiling of the merged GSE5406 and GSE57338 datasets was extracted for conducting WGCNA utilizing WGCNA package (Langfelder and Horvath, 2008). Sample clustering was implemented for testing whether there were outlier specimens. Soft threshold power value was determined for constructing a scale-free topology network. Afterwards, the adjacency matrix was converted to the topological overlap matrix (TOM). In accordance with the TOM-based dissimilarity, distinct co-expression modules were clustered. Associations of modules and RBP-based subtypes were then evaluated. The module with the strongest correlation to RBP-based subtypes was screened, and the genes in this module were regarded as RBP-based subtype-related genes.

### Protein-protein interaction analyses

RBP-based subtype-related genes were imported into the Search Tool for the Retrieval of Interacting Genes (STRING) online platform (https://www.string-db.org) (Szklarczyk et al., 2021). The interactions between their protein products were retrieved according to the default criteria. Utilizing MCODE plug-in of Cytoscape software (Doncheva et al., 2019), PPI subnetwork and hub genes were obtained following the selection criteria of degree cutoff = 2, node score cutoff = 0.2, haircut = true, Fluff = false, K-core = 2 Max, and depth from seed = 100.

### Selection of characteristic genes with two machine learning approaches

Characteristic RBP-based subtype-related genes were selected utilizing two machine learning approaches: least absolute shrinkage and selection operator regression (LASSO) as well as support vector machine recursive feature elimination (SVM-RFE). Through glmnet package (Engebretsen and Bohlin, 2019), LASSO was run and penalty parameter λ tuning was implemented using ten-fold cross-validation. Additionally, the best variables were selected with ten-fold cross-validation utilizing SVM-RFE algorithm. Afterwards, characteristic genes were determined through intersection of LASSO- and SVM-RFE-derived results.

### Establishment of a nomogram

A nomogram was established on the basis of characteristic genes *via* rms package. The accuracy of the nomogram in predicting risk probabilities was evaluated with calibration curve. Decision curve analyses represent a novel approach for assessing clinical usefulness, which were utilized to evaluate the clinical utility of the nomogram. Clinical impact curves were drawn for assessing the clinical usefulness and applicability net benefits of the nomogram with the optimal diagnostic value.

### Immune cell estimations

CIBERSORT (Newman et al., 2015) is an approach on the basis of the gene expression matrix for reliably estimating the relative abundance of 24 immune cell types in tissue specimens. CIBERSORT analyses were utilized for comparing differences in distinct immune cell types between groups. Spearman correlation analyses were implemented for exploring interactions between infiltrating immune cell types and characteristic genes.

### Connectivity map (CMap) analysis

Heart failure-specific RBPs were utilized to query the CMap database (https://clue.io/). Compounds with *p* < 0.05 were selected as potential therapeutic drugs for heart failure based upon transcriptome data. The mode of action (MoA) of these compounds was then analyzed.

### Statistical analyses

R software (www.r-project.org; version 3.6.1) was employed for all statistics analysis processes. Comparison analyses between groups were implemented utilizing Wilcoxon or Kruskal-Wallis test. The diagnostic efficacy of characteristic genes or nomogram was evaluated with receiver operator characteristic (ROC) curves along with area under the curve (AUC) calculation. Pearson or Spearmon correlation test was implemented for interactions between variables. *p* < 0.05 was considered statistically significant for all analysis process.

## Results

### Analyses of heart failure-specific RBPs

**FIGURE 1:**
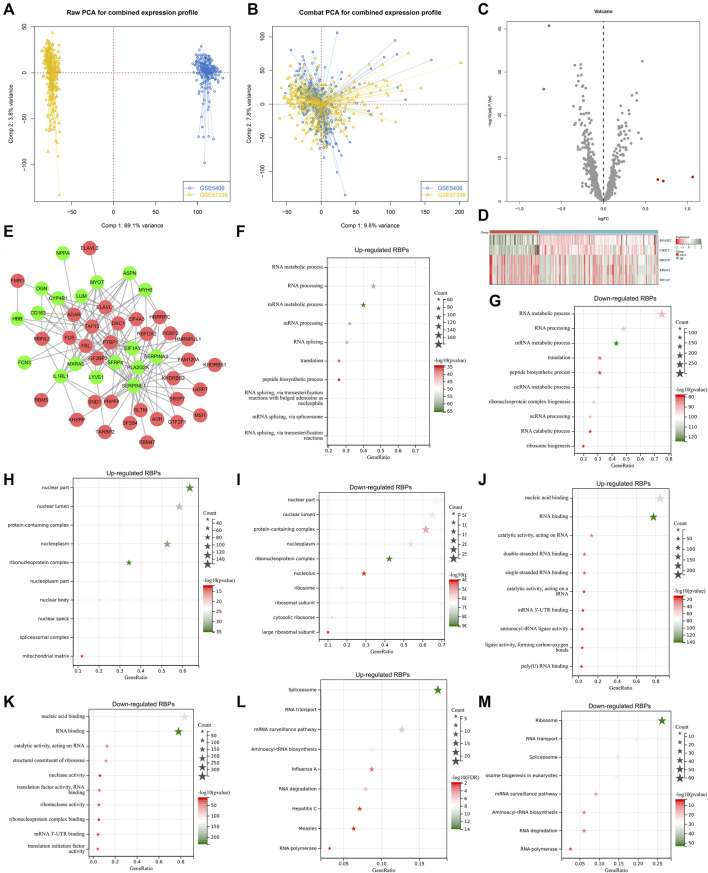
Analyses of heart failure-specific RBPs. **(A)** Integration of two heart failure expression profiling datasets (GSE5406, and GSE57338). **(B)** Removal of batch effects of the merged datasets. **(C)** Volcano plots of RBPs with differential expression between human failing and nonfailing control LV myocardium specimens following adjusted *p* < 0.05 and |fold-change|>1.5. **(D)** Heatmap of expression values of heart failure-specific RBPs in human failing (blue) and nonfailing control (red) LV myocardium specimens. **(E)** Potential downstream targets of RBPs with adjusted *p* < 0.05 that were differentially expressed in heart failure LV myocardium specimens than nonfailing controls. **(F–K)** The first ten biological processes, cellular components, and molecular functions of up- and down-regulated RBPs. **(L,M)** KEGG pathways enriched by up- and down-regulated RBPs.

Our study collected and merged two heart failure expression profiling datasets (GSE5406, and GSE57338), and batch effects were corrected for subsequent analyses (Figures 1A,B). To determine heart failure-specific RBPs, differential expression analyses were implemented. In accordance with adjusted *p* < 0.05 and |fold-change|>1.5, five RBPs (EIF1AY, RPS4Y1, DDX3Y, RNASE2, and CSDC2) were found in heart failure LV myocardium specimens in comparison to nonfailing controls (Figures 1C,D). Afterwards, we predicted potential downstream targets of RBPs with adjusted *p* < 0.05 through starBase database. As depicted in Figure 1E, 18 targets (SERPINA3, FCN3, LUM, ASPN, IL1RL1, SFRP4, CD163, MYOT, OGN, MXRA5, LYVE1, MYH6, PLA2G2A, CYP4B1, SERPINE1, HBB, NPPA, and EIF1AY) had the potential binding sites of RBPs, which were differentially expressed in heart failure LV myocardium specimens in contrast to nonfailing controls. Biological functions and pathways of RBPs with adjusted *p* < 0.05 were then probed. Biological processes such as RNA/mRNA/peptide/ncRNA metabolic processes, RNA processing, and translation were both remarkably linked to up- and down-regulated RBPs (Figures 1F,G), indicating their essential roles in modulating gene expression. Additionally, RBPs with adjusted *p* < 0.05 closely correlated to multiple cellular components (nuclear part and lumen, protein-containing complex, nucleoplasm, etc.), as illustrated in Figures 1H,I. Up- and down-regulated RBPs also possessed the molecular functions of nucleic acid binding, RNA binding, catalytic activity acting on RNA, etc. (Figures 1J,K). To probe signaling pathways involved in RBPs with adjusted *p* < 0.05, KEGG enrichment analyses were implemented. In Figures 1L,M, RNA transport/degradation/polymerase, spliceosome, mRNA surveillance pathway, etc. were remarkably enriched by up- and down-regulated RBPs.

### Establishment of RBP-based subtypes for heart failure

**FIGURE 2:**
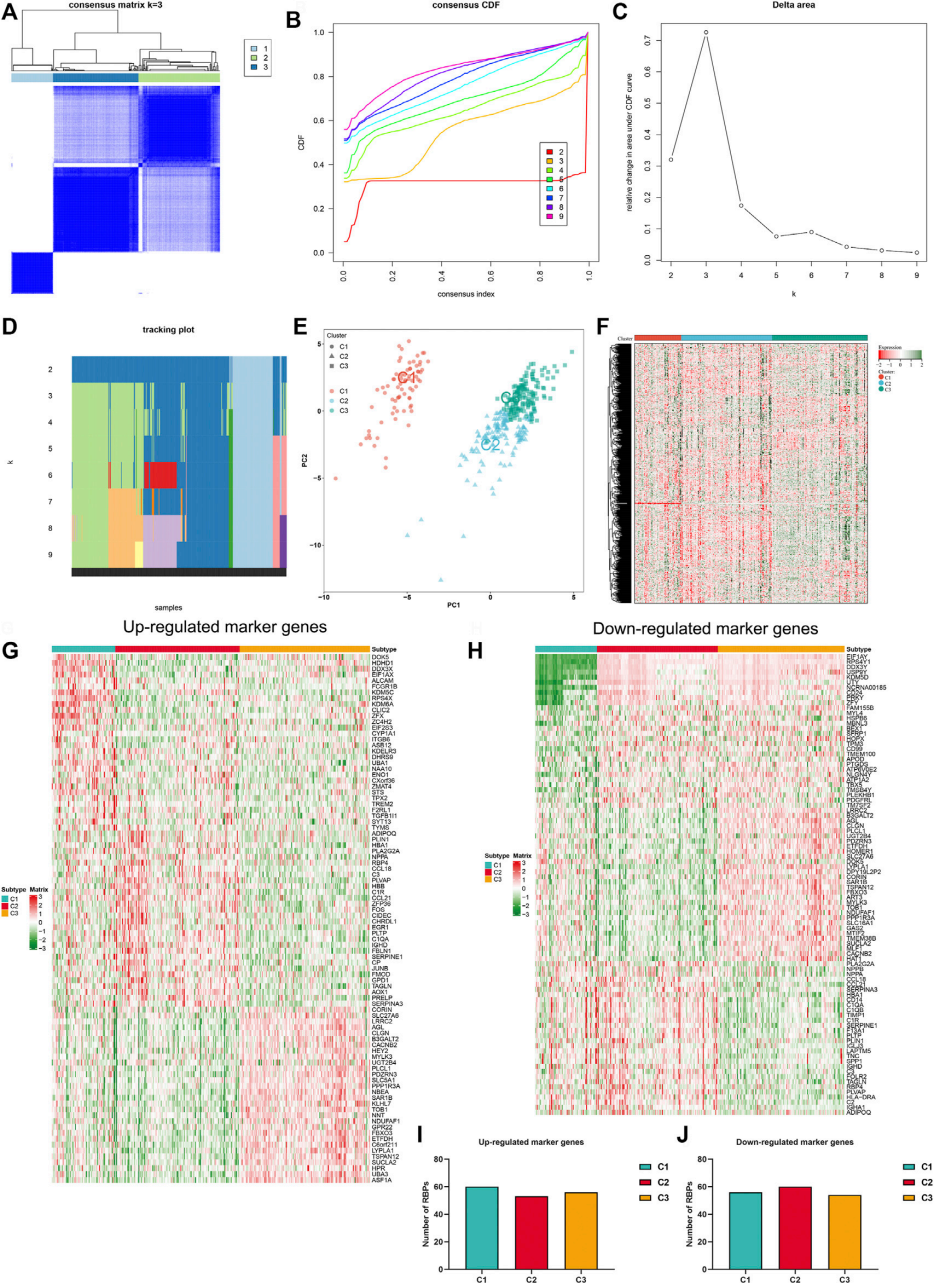
Establishment of RBP-based subtypes for heart failure. **(A)** Consensus matrix heatmap at *k* = 3. The rows and columns are samples, with consensus values on a white to blue color scale. **(B)** Consensus CDF curves at *k* = 2–9. **(C)** Delta area plot. **(D)** Tracking plot. The rows are samples, and the columns are k values. **(E)** PCA plot of three RBP-based subtypes. **(F)** Heatmap of expression values of RBPs with adjusted *p* < 0.05 across three subtypes. **(G)** The top 30 up-regulated marker genes in each subtype. **(H)** The top 30 down-regulated marker genes in each subtype. **(I,J)** Number of RBPs of up- and down-regulated marker genes in three subtypes.

Consensus clustering analyses were employed for identifying RBP-based subtypes across heart failure specimens in accordance with RBPs with adjusted *p* < 0.05. Figure 2A illustrated the consensus matrix heatmap at *k* = 3. We found that heart failure specimens could be clearly categorized as three RBP-based subtypes, namely C1, C2 and C3. Consistent cumulative distribution (CDF) plot showed that when *k* = 3, CDF reached an approximate maximum (Figure 2B). As depicted in delta area plot, when *k* = 4, the area under the CDF curve increased only slightly, and thus 3 was an appropriate k value (Figure 2C). Tracking plot was also established for visualizing the sample classification. When *k* = 3, this classification had relatively high stability (Figure 2D). By reason of the foregoing, three RBP-based subtypes were finally identified across heart failure samples. The accuracy of this classification was verified through PCA plot. In Figure 2E, heart failure samples were clearly classified as three subtypes. Additionally, RBPs with adjusted *p* < 0.05 presented different expression values across three RBP-based subtypes (Figure 2F). Differentially expressed genes among three RBP-based subtypes were analyzed, and we identified the top 30 up- or down-regulated genes in each subtype compared with others, which were considered as specific marker genes of each subtype (Figures 2G,H). Potential RBPs of up- and down-regulated marker genes were predicted, respectively. As illustrated in Figures 2I,J, no notable differences in number of RBPs of up- and down-regulated marker genes were found across three subtypes.

### Different molecular mechanisms across RBP-based subtypes

**FIGURE 3:**
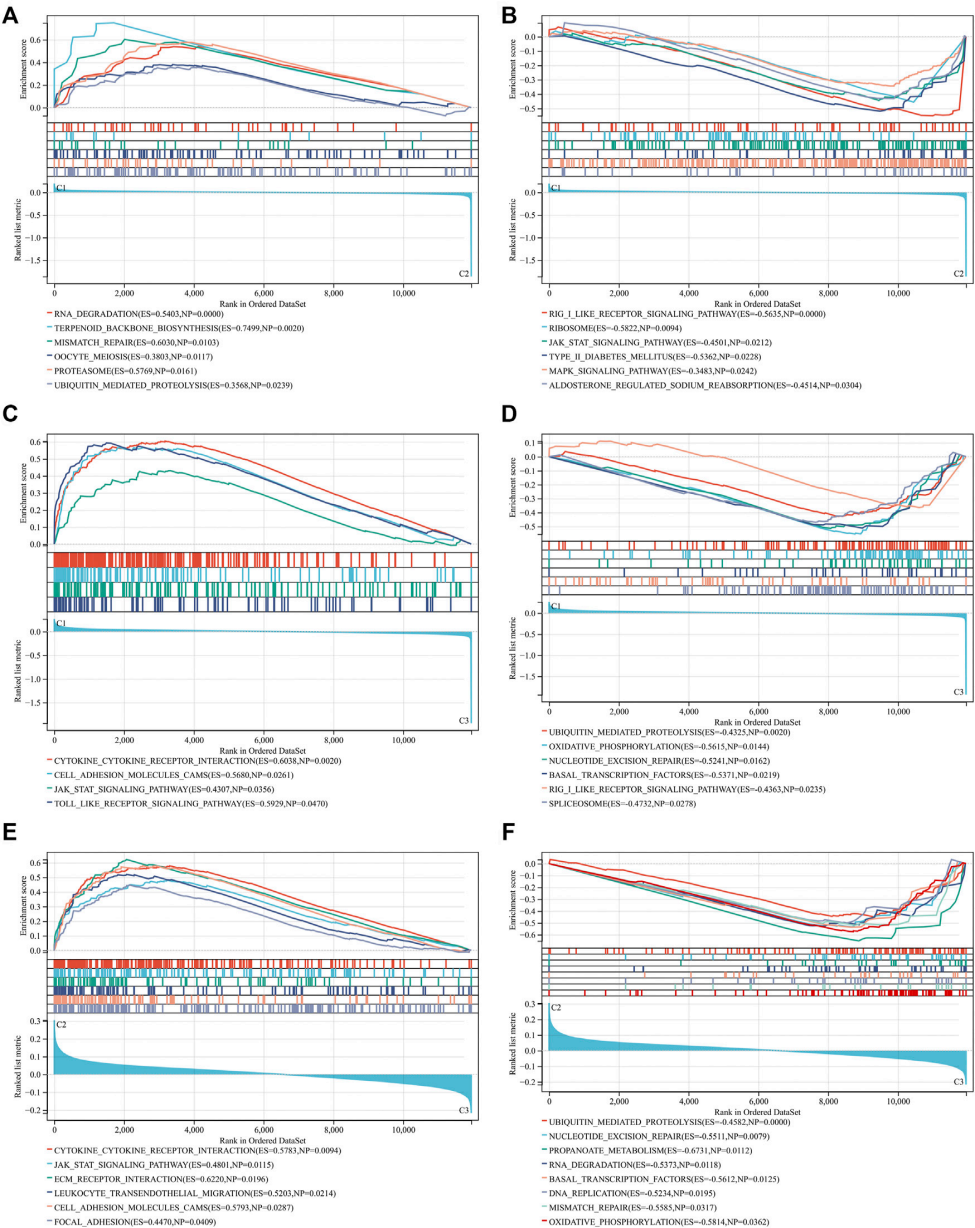
Different molecular mechanisms across RBP-based subtypes. **(A,B)** GSEA for comparing KEGG pathways between C1 and C2 subtypes. **(C,D)** GSEA for comparing KEGG pathways between C1 and C3 subtypes. **(E,F)** GSEA for comparing KEGG pathways between C2 and C3 subtypes.

Through GSEA, we dissected the differences in molecular mechanisms between distinct RBP-based subtypes. Compared to C2 subtype, RNA degradation, terpenoid backbone biosynthesis, mismatch repair, oocyte meiosis, proteasome, and ubiquitin mediated proteolysis were remarkably activated in C1 subtype (Figure 3A). Meanwhile, activation of RIG I like receptor signaling pathway, ribosome, JAK-STAT signaling pathway, type II diabetes mellitus, MAPK signaling pathway and aldosterone regulated sodium reabsorption was found in C2 subtype (Figure 3B). Molecular mechanisms between C1 and C3 subtypes were then compared. In Figure 3C, C1 subtype presented the remarkable activation of cytokine-cytokine receptor interaction, cell adhesion molecules CAMS, JAK-STAT signaling pathway and Toll-like receptor signaling pathway than C3 subtype. In comparison to C1 subtype, ubiquitin mediated proteolysis, oxidative phosphorylation, nucleotide excision repair, basal transcription factors, RIG I like receptor signaling pathway, and spliceosome were significantly activated in C3 subtype (Figure 3D). Additionally, we found the significant activation of cytokine-cytokine receptor interaction, JAK-STAT signaling pathway, ECM receptor interactions, leukocyte trans-endothelial migration, cell adhesion molecules CAMS, and focal adhesion in C2 subtype in comparison to C3 subtype (Figure 3E). Meanwhile, ubiquitin mediated proteolysis, nucleotide excision repair, propanoate metabolism, RNA degradation, basal transcription factors, DNA replication, mismatch repair, and oxidative phosphorylation were markedly activated in C3 subtype (Figure 3F). Above data indicated the distinct molecular mechanisms across RBP-based subtypes.

### Distinct PANoptosis features across RBP-based subtypes

**FIGURE 4:**
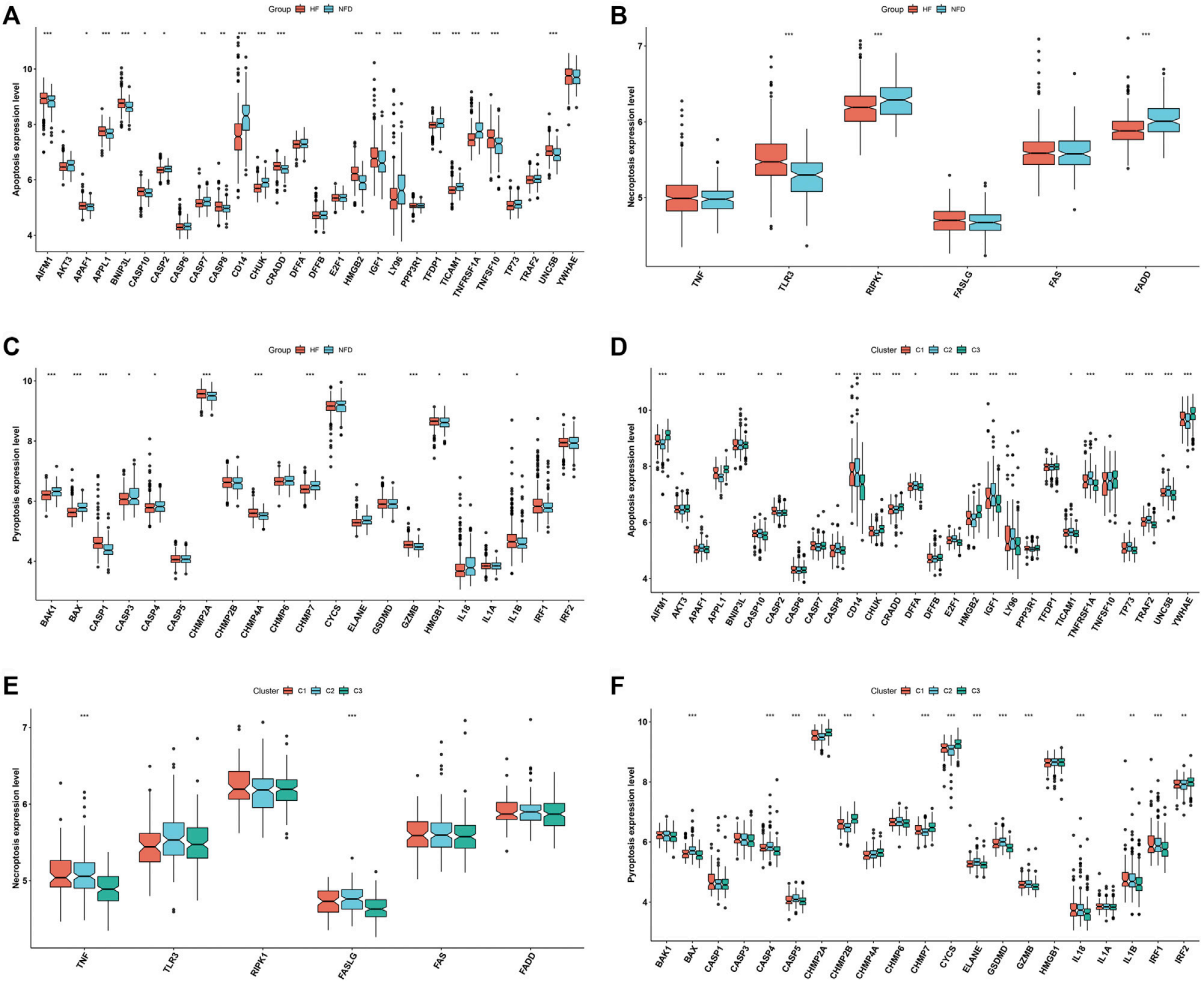
Distinct PANoptosis features across RBP-based subtypes. **(A–C)** Levels of apoptosis, necroptosis and pyroptosis genes in human failing and nonfailing control LV myocardium specimens. **(D–F)** Levels of apoptosis, necroptosis and pyroptosis genes across three RBP-based subtypes (*p < 0.05; **p < 0.01; ***p < 0.001).

Next, we focused on PANoptosis features in heart failure. Deregulation of PANoptosis (apoptosis, necroptosis and pyroptosis) genes was found in heart failure LV myocardium specimens *versus* nonfailing controls (Figures 4A–C), indicating that PANoptosis might be linked to heart failure. In addition, PANoptosis features were assessed across three RBP-based subtypes. The widespread heterogeneity in PANoptosis (apoptosis, necroptosis and pyroptosis) genes was observed across RBP-based subtypes (Figures 4D–F).

### Identification of RBP-based subtype-related genes

**FIGURE 5:**
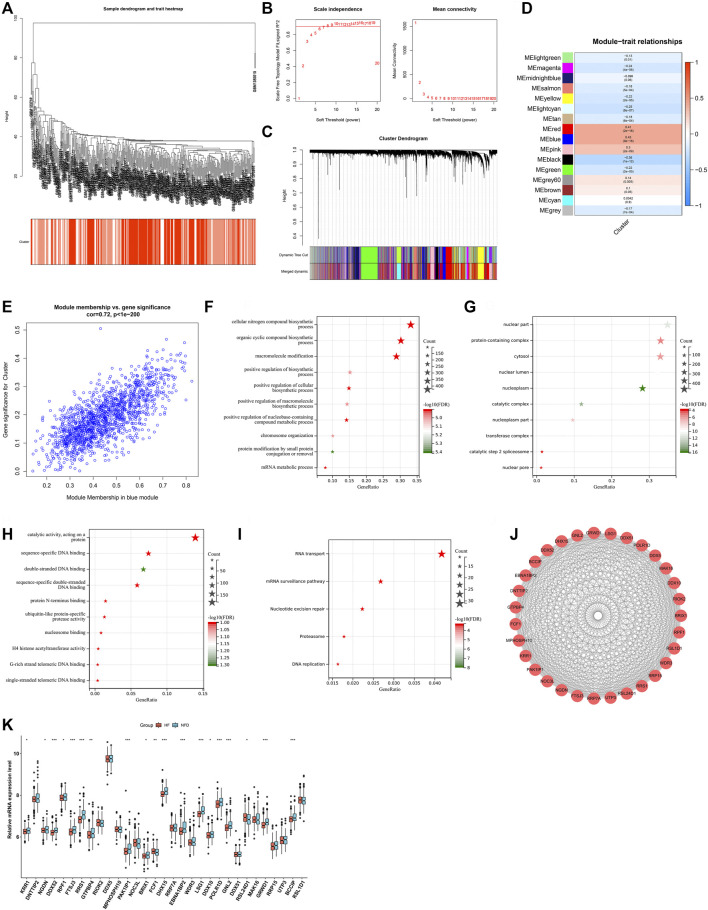
Identification of RBP-based subtype-related genes. **(A)** Clustering dendrogram of heart failure specimens on the basis of Euclidean distance. **(B)** Scale independence and mean connectivity at different power values. **(C)** Cluster dendrogram of distinct modules through dynamic tree cut approach and merged dynamic approach. The gray module represents unclassified genes. **(D)** Correlations between modules and RBP-based subtypes. The color indicates the strength of the correlation, and the number in parentheses indicates *p*-value. **(E)** Scatter plot of the relationship between module membership of blue module and gene significance for RBP-based subtypes. **(F–H)** The first ten biological processes, cellular components, and molecular functions of RBP-based subtype-related genes. **(I)** KEGG pathways significantly linked to RBP-based subtype-related genes. **(J)** The PPI subnetwork of key RBP-based subtype-related genes. **(K)** Box plot of the expressions of key RBP-based subtype-related genes in human failing and nonfailing control LV myocardium specimens. *p < 0.05; **p < 0.01; ***p < 0.001.

WGCNA approach was utilized for determining RBP-based subtype-related genes. Hierarchical clustering analyses demonstrated no outlier specimens (Figure 5A). Soft-thresholding power is an import process of WGCNA. To establish a scale-free co-expression network, soft-thresholding power was set as 9 following scale independence and mean connectivity (Figure 5B). Genes were clustered through dynamic tree cut approach to obtain 16 modules (Figure 5C). Associations between modules and RBP-based subtypes were then evaluated. As a result, blue module presented the strongest correlation to RBP-based subtypes (Figure 5D). Additionally, we found the remarkable correlation between module membership of blue module and gene significance for RBP-based subtypes (Figure 5E). Thus, 1,460 genes in blue module were regarded as RBP-based subtype-related genes. Their biological functions and pathways were then probed. In Figure 5F, RBP-based subtype-related genes were remarkably linked to biosynthetic process. Also, they presented the associations with cellular components such as nuclear part, protein-containing complex, cytosol (Figure 5G). In Figure 5H, they possessed the molecular functions of catalytic activity acting on a protein, sequence-specific DNA binding, and double-stranded DNA binding, etc. RNA transport, mRNA surveillance pathway, nucleotide excision repair, proteasome and DNA replication were remarkably enriched by RBP-based subtype-related genes (Figure 5I). Above data proved their key roles in pathophysiologic processes of heart failure. Through MCODE approach, 31 key RBP-based subtype-related genes were selected, comprising KRR1, DNTTIP2, NGDN, DDX52, RPF1, FTSJ3, RRS1, GTPBP4, RIOK2, DDX5, MPHOSPH10, PAK1IP1, NOC3L, BRIX1, FCF1, DHX15, RRP7A, EBNA1BP2, WDR3, LSG1, DDX10, POLR1D, GNL2, DDX51, RSL24D1, MAK16, GRWD1, RRP15, UTP3, BCCIP, RSL1D1 (Figure 5J). Most key RBP-based subtype-related genes presented the down-regulation in failing than nonfailing control LV myocardium specimens (Figure 5K).

### Identification of characteristic genes for heart failure *via* machine learning analyses

**FIGURE 6:**
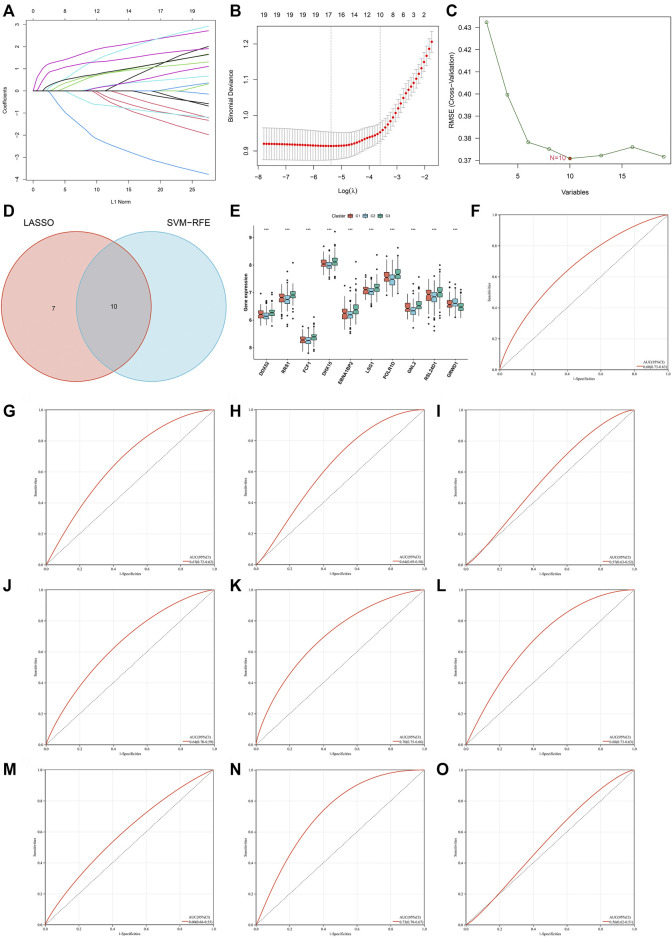
Identification of characteristic genes for heart failure *via* machine learning analyses. **(A)** Relationships between log-transformed lambda and regression coefficients. Each line indicates a variable. **(B)** LASSO regression profiling. The line represents 95% CI, and the dotted line represents the optimal number of variables. **(C)** Selection of characteristic genes through SVM-RPE approach. **(D)** Venn plot of characteristic genes shared by LASSO and SVM-RPE approaches. **(E)** Box plot of the expression of characteristic genes across three RBP-based subtypes (***p < 0.001). **(F–O)** ROC curves for assessing the predictive efficacy of characteristic genes: **(F)** DDX52, **(G)** DHX15, **(H)** EBNA1BP2, **(I)** FCF1, **(J)** GNL2, **(K)** GRWD1, **(L)** LSG1, **(M)** POLR1D, **(N)** RRS1, and **(O)** RSL24D1.

Two machine learning approaches LASSO and SVM-RPE were employed for selecting characteristic genes among RBP-based subtype-related genes. 17 and 10 characteristic genes were separately selected by LASSO (Figures 6A,B) and SVM-RPE (Figure 6C) methods. After intersection, ten characteristic genes were finally determined, including DDX52, DHX15, EBNA1BP2, FCF1, GNL2, GRWD1, LSG1, POLR1D, RRS1, and RSL24D1 (Figure 6D). C3 subtype presented the highest expressions of above characteristic genes, followed by C1 and C2 (Figure 6E). To assess the predictive efficacy of characteristic genes, ROC curves were plotted. As illustrated in Figures 6F–O, the AUC values (95%CI) of DDX52, DHX15, EBNA1BP2, FCF1, GNL2, GRWD1, LSG1, POLR1D, RRS1, and RSL24D1 were 0.68 (0.73–0.63), 0.67 (0.72–0.62), 0.64 (0.69–0.58), 0.57 (0.63–0.52), 0.64 (0.70–0.59), 0.70 (0.75–0.66), 0.68 (0.73–0.63), 0.60 (0.66–0.55), 0.73 (0.78–0.67), 0.56 (0.62–0.51), proving the excellent performance in diagnosing heart failure.

### Establishment of a characteristic gene-based nomogram for heart failure

**FIGURE 7:**
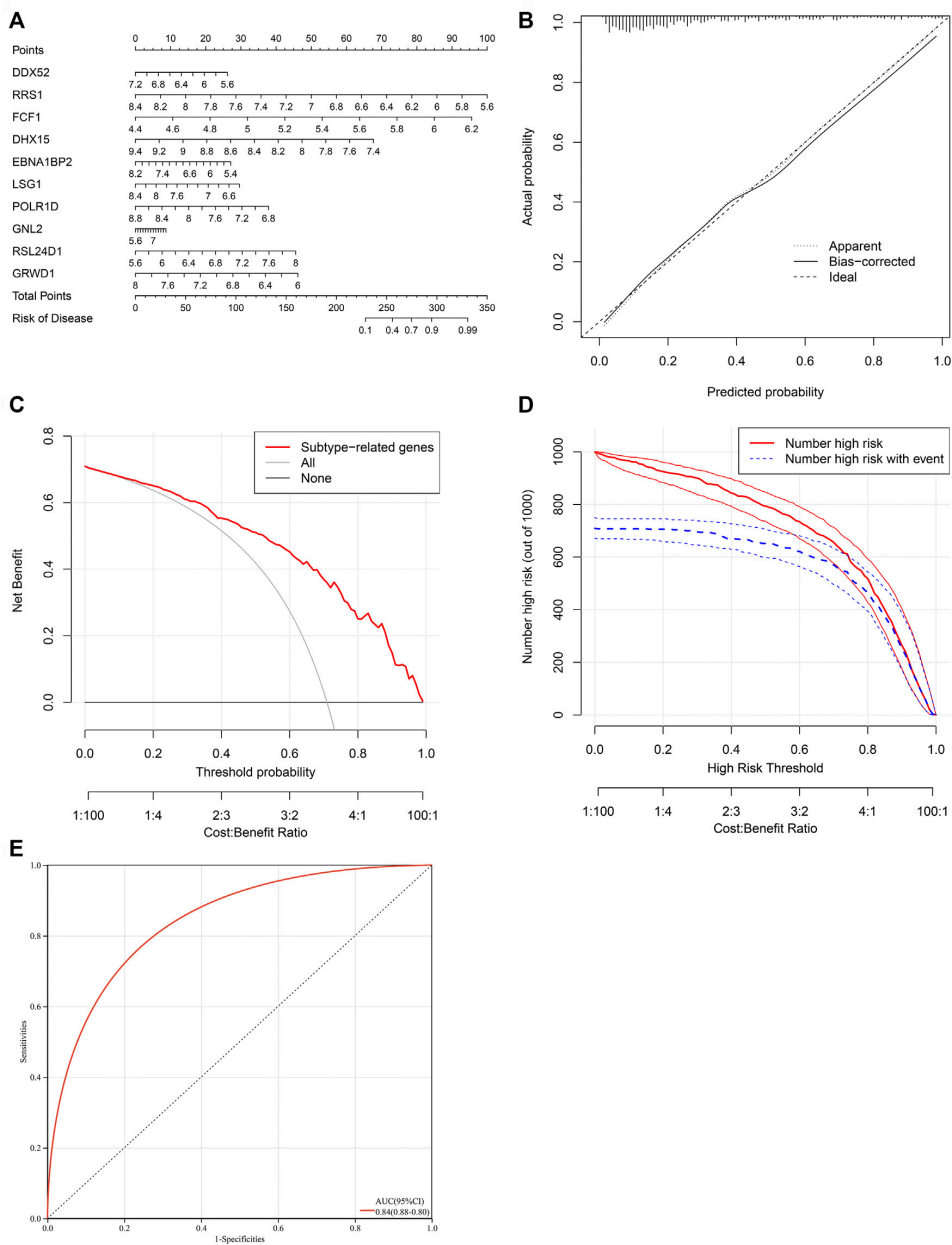
Establishment of a characteristic gene-based nomogram for heart failure. **(A)** A nomogram comprising characteristic genes for predicting risk of heart failure. **(B)** Calibration curve for actual and nomogram-predicted probability of heart failure. **(C)** Decision curve analyses for the net benefit curves of the nomogram. X-axis shows the threshold probability for heart failure and Y-axis represents the net benefit. **(D)** Clinical impact curves of the nomogram. Red curves indicate the number of patients classified as positive (high risk) by the nomogram at different threshold probabilities. Blue curves show the number of true positives at different threshold probabilities. **(E)** ROC curve for estimating the predictive efficacy of the nomogram.

To facilitate the clinical performance of characteristic genes, a nomogram was established for heart failure (Figure 7A). As illustrated in calibration curve, the nomogram-predicted risk probabilities were close to the actual probabilities of heart failure (Figure 7B). Decision curve analyses demonstrated that the nomogram possessed the preferred prediction efficacy, with the higher net benefit (Figure 7C). Clinical impact curves were drawn for evaluating clinical applicability of the risk predictive nomogram. As illustrated in Figure 7D, the nomogram showed the superior overall net benefit within the wide and practical ranges of threshold probabilities and influenced patients’ outcome, indicating that the nomogram possessed excellent predictive performance. Above data proved that the nomogram was clinically useful. Moreover, the AUC value (95%CI) of the nomogram was 0.84 (0.88–0.80) (Figure 7E), which was higher than any one of characteristic genes, demonstrating that the predictive efficacy of the nomogram was better compared with a single characteristic gene.

### Landscape of immune cells and PANoptosis features in heart failure

**FIGURE 8:**
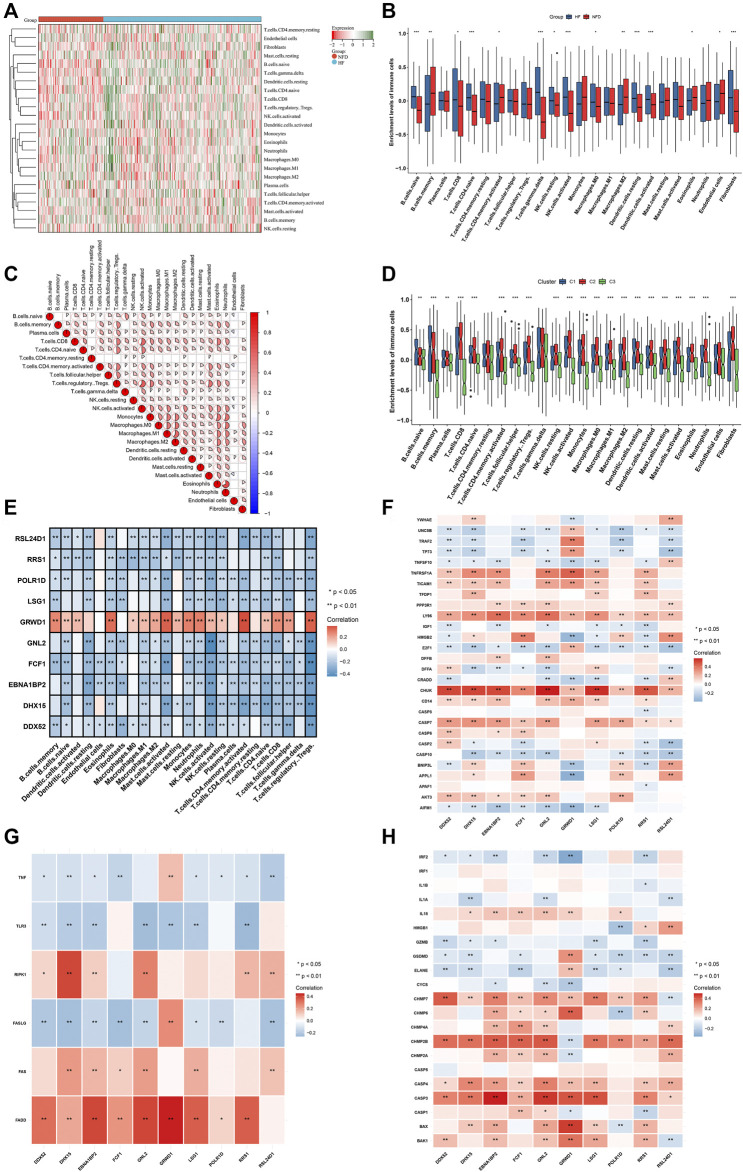
Landscape of immune cells and PANoptosis features in heart failure. **(A,B)** The abundance of 24 immune cell types in human failing (blue) and nonfailing control (red) LV myocardium specimens. **(C)** Associations between distinct immune cell types. **(D)** The abundance of 24 immune cell types across three RBP-based subtypes. **(E)** Associations between 24 immune cell types and characteristic genes. **(F–H)** Associations of characteristic genes with apoptosis, necroptosis and pyroptosis genes. *p < 0.05; **p < 0.01; ***p < 0.001.

CIBERSORT was employed for estimating the abundance of 24 immune cell types. Firstly, the abundance of immune cell types was compared between human failing and nonfailing control LV myocardium specimens. As illustrated in Figures 8A,B, failing myocardium tissues presented the enhanced abundance of B cell naïve, T cells CD8, T cells CD4 naïve, T cells gamma delta, NK cells resting/activated, macrophages M0, dendritic cells resting/activated, and fibroblasts. Meanwhile, the reduced abundance of B cells memory, T cells CD4 memory activated, macrophages M2, eosinophils, endothelial cells was found in failing myocardium. Additionally, the positive interactions across immune cell types were found, as illustrated in Figure 8C. We also assessed the differences in immune cell types across three RBP-based subtypes. Generally, C2 had the highest abundance of most immune cells, followed by C1 and C3 (Figure 8D). Figure 8E depicted the associations between characteristic genes and immune cell infiltration. Most characteristic genes were negatively linked to the abundance of immune cells, but GRWD1 presented the positive interactions with most immune cell types. In addition, characteristic genes exhibited notable associations with PANoptosis (apoptosis, necroptosis and pyroptosis) genes (Figures 8F–H).

### External verification of characteristic genes in heart failure

**FIGURE 9:**
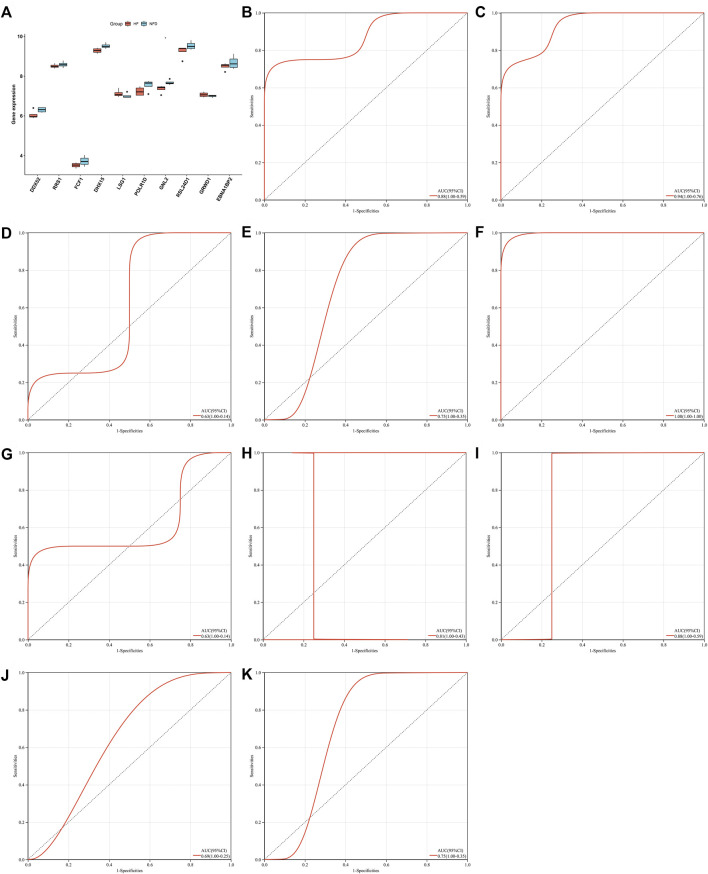
External verification of characteristic genes in heart failure in the GSE76701 dataset. **(A)** Box plot of the expressions of characteristic genes in human failing (red) and nonfailing control (blue) LV myocardium specimens (*p < 0.05). **(B–K)** Evaluation of diagnostic performance of **(B)** DDX52, **(C)** DHX15, **(D)** EBNA1BP2, **(E)** FCF1, **(F)** GNL2, **(G)** GRWD1, **(H)** LSG1, **(I)** POLR1D, **(J)** RRS1, and **(K)** RSL24D1 in heart failure through ROC curves.

Characteristic genes in heart failure were externally verified in independent datasets. In Figure 9A, DDX52, RRS1, FCF1, DHX15, POLR1D, GNL2, RSL24D1, and EBNA1BP2 presented the low expressions in failing than nonfailing control heart. Inversely, LSG1, and GRWD1 expressions were up-regulated in failing compared with nonfailing control heart in the GSE76701 dataset. The abnormal expression of characteristic genes between human failing and nonfailing control LV myocardium tissues was confirmed in the GSE55296 dataset (Supplementary Figure S1). ROC curves were conducted for evaluating the diagnostic efficacy of above characteristic genes in heart failure in the GSE76701 dataset. The AUC values (95%CI) of DDX52, DHX15, EBNA1BP2, FCF1, GNL2, GRWD1, LSG1, POLR1D, RRS1, and RSL24D1 were 0.88 (1.00–0.59), 0.94 (1.00–0.76), 0.63 (1.00–0.14), 0.75 (1.00–0.35), 1.00 (1.00–1.00), 0.63 (1.00–0.14), 0.81 (1.00–0.43), 0.88 (1.00–0.59), 0.69 (1.00–0.25), and 0.75 (1.00–0.35), as illustrated in Figures 9B–K. However, no significant differences in characteristic genes were observed between HFpEF and HFrEF heart tissues in the GSE86569 dataset (Supplementary Figure S2). In addition, the GSE3585 dataset was adopted to validate the diagnostic efficacy of the characteristic genes. The excellent diagnostic performance of each characteristic gene was proven, as shown in Supplementary Figures S3A–H. Above evidence confirmed that characteristic genes enabled to excellently diagnose heart failure.

### Prediction of potential therapeutic compounds of heart failure

**FIGURE 10:**
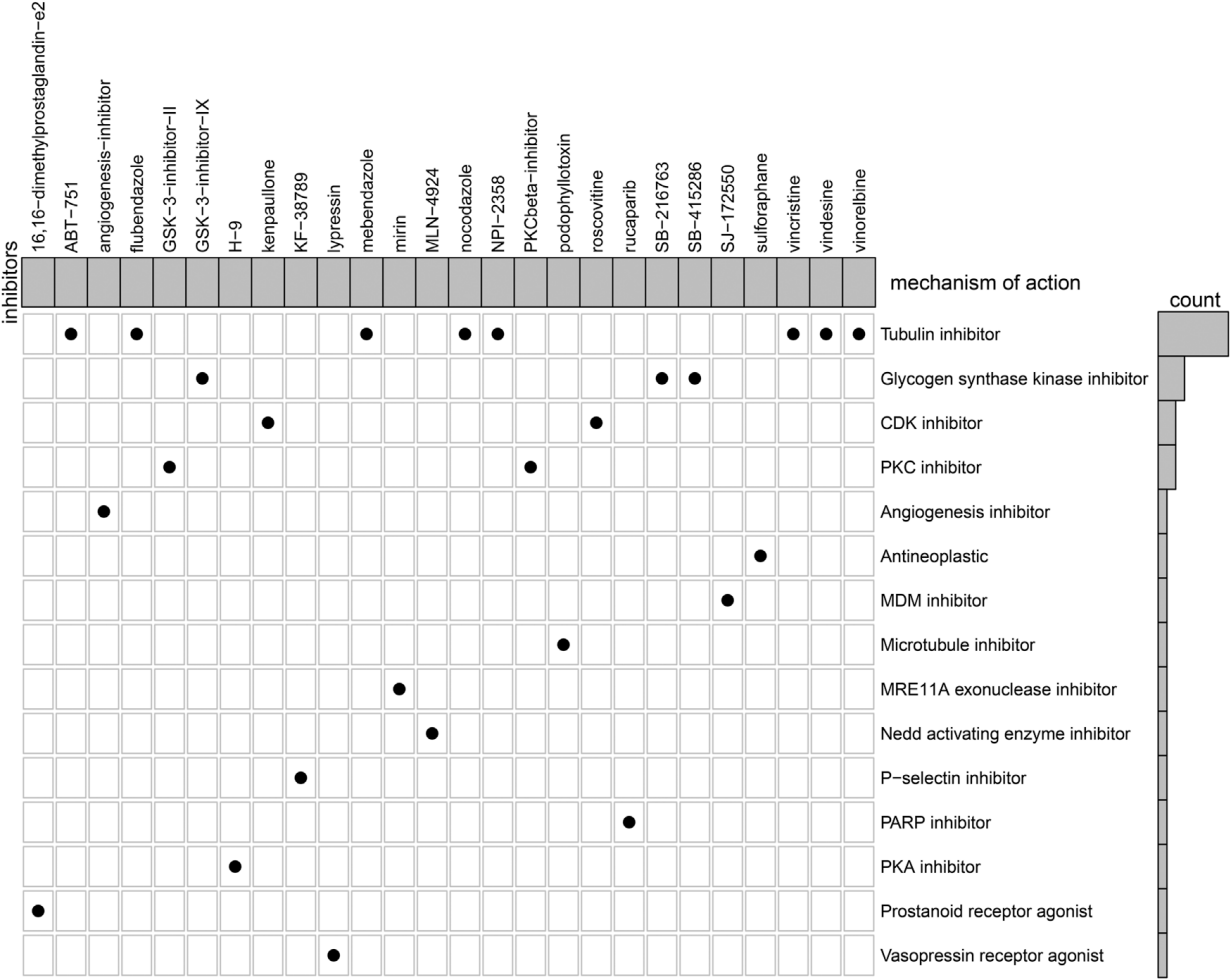
MoA analysis for the shared mechanisms of potential therapeutic compounds of heart failure.

Based on heart failure-specific RBPs, CMap analysis was adopted to screen potential compounds for the treatment of heart failure with *p* < 0.05. In accordance with MoA analysis, mebendazole, NPI-2358, vindesine, vincristine, flubendazole, vinorelbine, nocodazole, and ABT-751 shared tubulin inhibitor (Figure 10). GSK-3-inhibitor-IX, SB-415286, and SB-216763 shared glycogen synthase kinase inhibitor. Roscovitine and kenpaullone shared CDK inhibitor. GSK-3-inhibitor-II and PKCbeta-inhibitor shared PKC inhibitor.

## Discussion

RBPs have been described to be expressed and modulated in a variety of organs especially human heart (Gupta et al., 2018). Despite this, little is known concerning the roles of RBPs in heart failure. Thus, our study implemented comprehensive analyses of RBPs in heart failure, and determined RBP-based subtypes, and RBP-based subtype-related characteristic genes, unveiling the crucial functions of RBPs in heart failure.

RBPs are crucial effectors of gene expression, and as such their abnormal expressions underlie the origin of heart failure (Gebauer et al., 2021). On the basis of the merged GSE5406, and GSE57338 expression profiling datasets, we determined five RBPs (EIF1AY, RPS4Y1, DDX3Y, RNASE2, and CSDC2) with adjusted *p* < 0.05 and |fold-change|>1.5 in heart failure LV myocardium specimens than nonfailing controls, which were regarded as hear failure-specific RBPs. RBPs with adjusted *p* < 0.05 were closely linked to RNA metabolism processes (RNA/mRNA/peptide/ncRNA metabolic processes, RNA splicing, and translation) as well as pathways (RNA transport, mRNA surveillance pathway, ribosome biogenesis in eukaryotes, aminoacyl-tRNA biosynthesis, RNA degradation, etc.), highlighting the crucial functions of RBPs in controlling gene expression. Evidence has demonstrated that deregulation of RNA metabolism leads to heart failure progression (Kim et al., 2018). On the basis of RBPs with adjusted *p* < 0.05, three RBP-based subtypes were established, characterized by distinct signaling pathway activities. Additionally, RBP-based subtype-related genes were further determined, which might be modulated by RBPs.

Previous studies have determined heart-specific RBPs (RBM20, RBM24, HuR, etc.) that were not included in our heart failure-specific RBPs. For instance, suppressing RBM20 activity may improve diastolic dysfunction and cardiac atrophy (Hinze et al., 2016). RBM24 loss destroys global alternative splicing and contributes to dilated cardiomyopathy (Liu et al., 2019). HuR-induced SCN5A mRNA stability decreases arrhythmic risk in heart failure (Zhou et al., 2018). Thus, our study offered novel heart-specific RBPs. More experiments are awaited to validate the biological functions of heart failure.

LASSO is a regression analysis approach that utilizes regularization for improving the predictive accuracy (Xing et al., 2022). SVM-RFE is a reliable feature selection approach that determines the optimal variables through removing the feature vectors produced by SVM (Zhao et al., 2020). Through integrating two machine learning approaches, ten characteristic genes were eventually determined, comprising DDX52, DHX15, EBNA1BP2, FCF1, GNL2, GRWD1, LSG1, POLR1D, RRS1, and RSL24D1. All of them accurately predicted the risk of heart failure. Further, a characteristic gene-based nomogram was established, which was capable of accurately predicting heart failure risk, with the excellent clinical usability. DDX52 is a type of DEAD/H box RNA helicase, and its suppression exerts an anti-tumor effect (Yu et al., 2021). The DEAH-box RNA helicase DHX15 has been identified as a potential gene for pathological cardiac hypertrophy triggered by excessive exercise (Zhou et al., 2020) and pulmonary arterial hypertension (Wang et al., 2021). EBNA1BP2 functions as a dynamic scaffold for ribosome biogenesis (Hirano et al., 2009). FCF1 is a potential marker of circulating breast cancer cells for detecting metastasis (Fina et al., 2022). The nucleolar GTP-binding protein GNL2 is essential for retinal neurogenesis in developing zebrafish (Paridaen et al., 2011). Cdt1-binding protein GRWD1 acts as a histone-binding protein, which triggers MCM loading *via* influencing chromatin architecture (Sugimoto et al., 2015). LSG1 is a family member of essential GTPases, in relation to the evolution of compartmentalization (Reynaud et al., 2005). POLR1D is a component of RNA polymerase I and RNA polymerase III complexes, mediating the synthesis of ribosomal RNA precursor and small RNA (Sanchez et al., 2020). RRS1 is a key factor of 5 S rRNA binding activity (Kharde et al., 2015). RSL24D1 participates in the biogenesis of the 60 S ribosomal subunit (Ni et al., 2022).

Heart failure is typically linked to cardiac remodeling, and inflammatory response plays a crucial role. During cardiac inflammation, immune cells invade the cardiac tissue as well as modulate tissue-damaging response (Bacmeister et al., 2019). In the present study, failing myocardium tissues exhibited the enhanced abundance of B cell naïve, T cells CD8, T cells CD4 naïve, T cells gamma delta, NK cells resting/activated, macrophages M0, dendritic cells resting/activated, and fibroblasts. Additionally, the decreased abundance of B cells memory, T cells CD4 memory activated, macrophages M2, eosinophils, endothelial cells was observed in failing myocardium. For example, CD8^+^ effector T cells may prevent cardioprotective macrophage differentiation in early heart failure (Komai et al., 2021). Posttranscriptional control of mRNA modulates inflammatory and immune responses. Several RBPs have been extensively explored, and bind target mRNAs to enhance or dampen above activities (Akira and Maeda, 2021). RBP-based C2 subtype presented the highest abundance of most immune cells, followed by C1 and C3. In addition, there was the extensive heterogeneity in PANoptosis traits across three RBP-based subtypes. Most characteristic genes presented negative correlations to the abundance of immune cells in heart failure, but GRWD1 was positively linked to most immune cell types, indicating their functions in mediating cardiac inflammation. Among them, evidence demonstrates that DHX15 may sense double-stranded RNA in myeloid dendritic cells to activate the immune response to RNA (Lu et al., 2014). Co-expression network analyses have determined DHX15 RNA helicase as a regulator of B cells (Detanico et al., 2019). DHX15 is a crucial regulator of natural killer-cell homeostasis and function (Wang et al., 2022). Additionally, characteristic genes exhibited remarkable interactions to PANoptosis features across heart failure. More experiments are required for verifying their regulatory functions in inflammatory and immune responses as well as PANoptosis in heart failure.

## Conclusion

Collectively, our findings provided an overview of RBPs involved in heart failure. Three RBP-based subtypes as well as ten relevant characteristic genes were determined for heart failure, elucidating the critical roles of RBPs in pathophysiological process (especially immunity and PANoptosis) of heart failure as well as offering reliable targets for diagnosing heart failure risk. Despite this, in-depth research is required for verifying the effectiveness of RBP-based subtypes and characteristic genes in diagnostic utility of heart failure.

## Data Availability

The original contributions presented in the study are included in the article/Supplementary Material, further inquiries can be directed to the corresponding authors.

## Abbreviations

LVEF: left ventricular ejection fraction; RBPs: RNA binding proteins; GEO: Gene Expression Omnibus; LV: left ventricular; GO: Gene Ontology; KEGG: Kyoto Encyclopedia of Genes and Genomes; PCA: principal component analyses; GSEA: Gene set enrichment analyses; WGCNA: weighted gene co-expression network analyses; TOM: topological overlap matrix; PPI: protein-protein interaction; STRING: Search Tool for the Retrieval of Interacting Genes; LASSO: least absolute shrinkage and selection operator regression; SVM-RFE: support vector machine recursive feature elimination; ROC: receiver operator characteristic; AUC: area under the curve; CMap: Connectivity Map.
