# Supplementary figures and images for: Landscape of RNA-binding proteins in diagnostic utility, immune cell infiltration and PANoptosis features of heart failure
Source: Front Genet. 2022 Oct 14;13:1004163. doi: 10.3389/fgene.2022.1004163 (PMC9614340; doi:10.3389/fgene.2022.1004163)

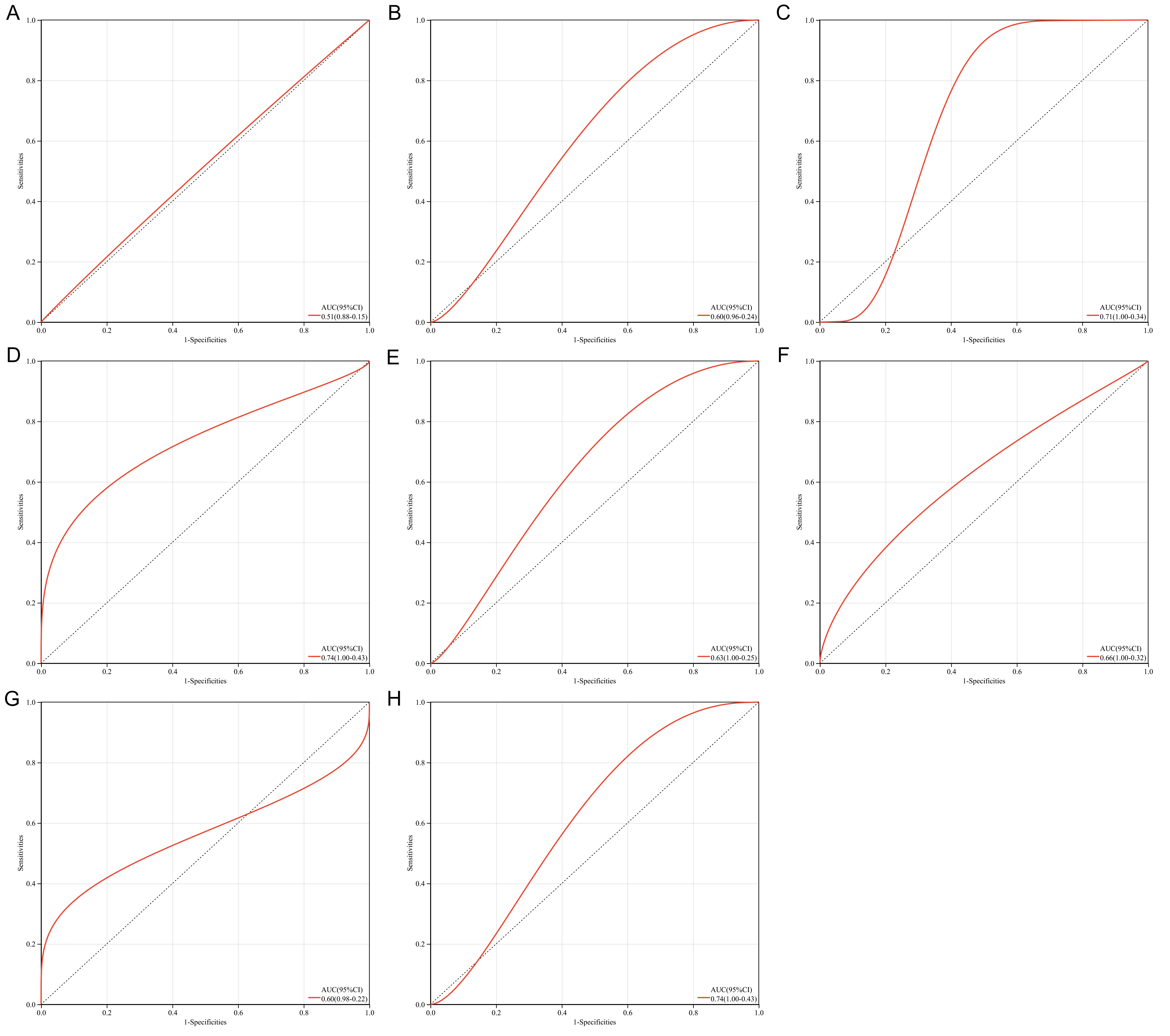

Supplement: Supplementary file 1 [file Image3.TIF]

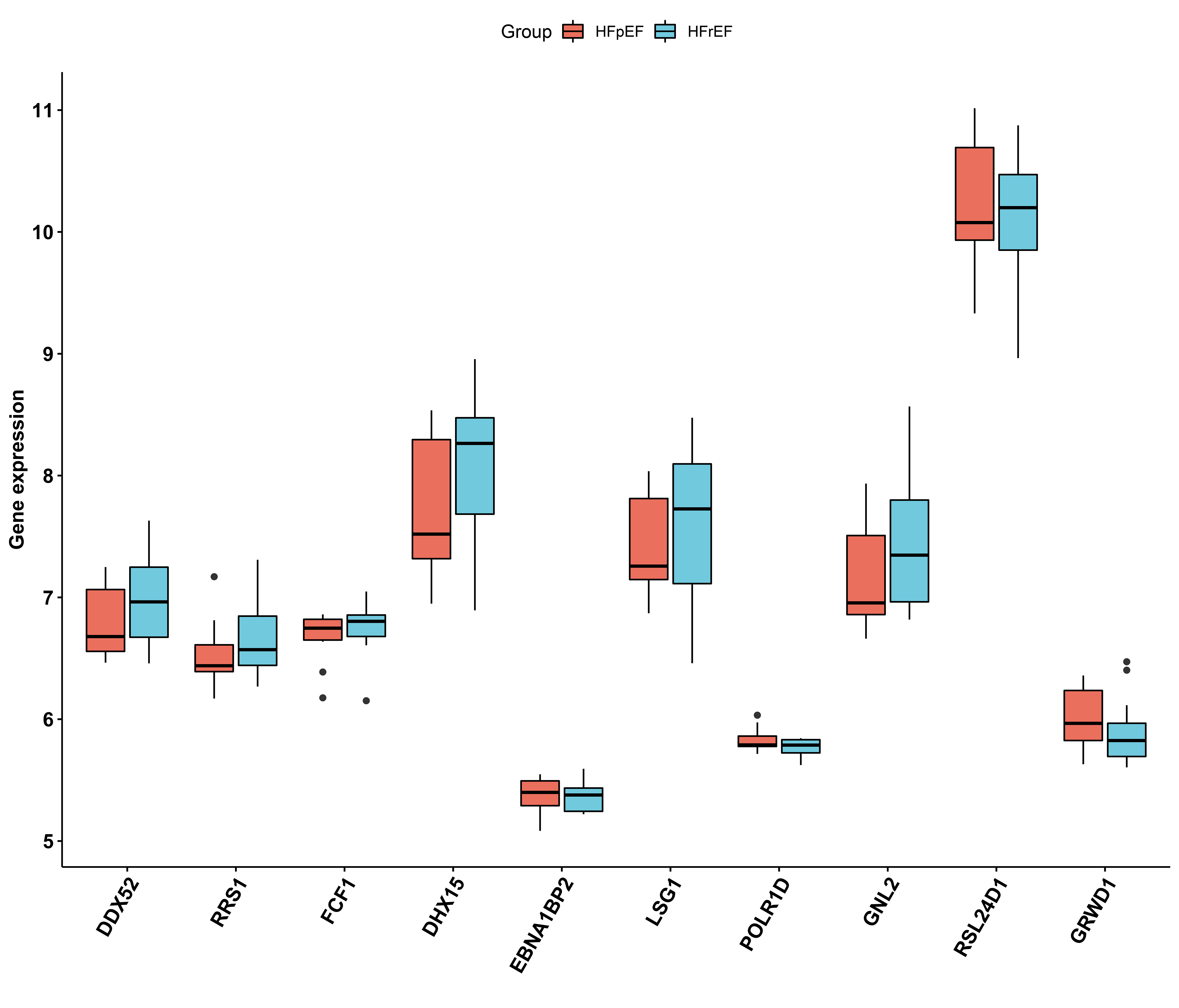

Supplement: Supplementary file 2 [file Image2.TIF]

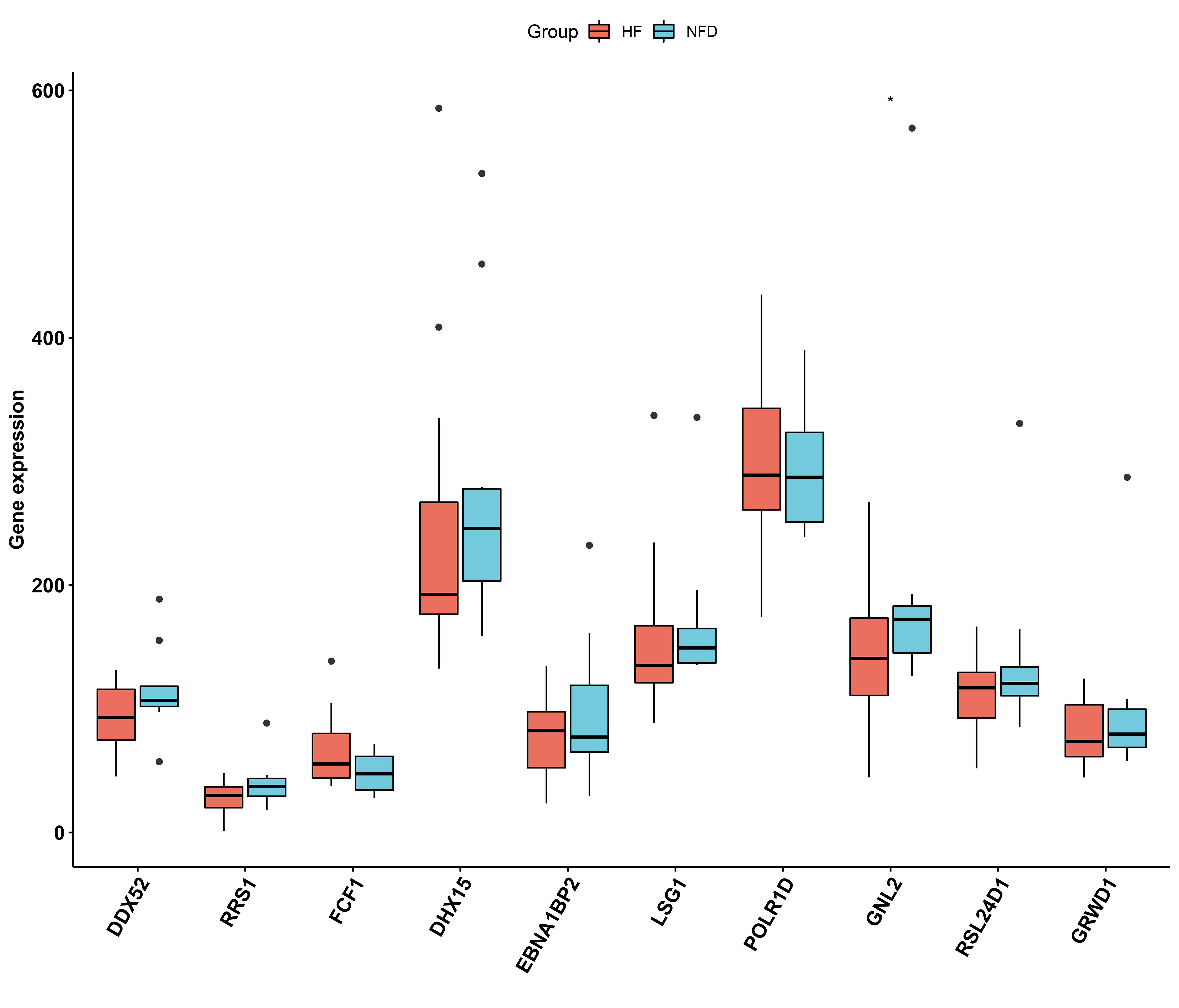

Supplement: Supplementary file 3 [file Image1.TIF]
